# Socio-cultural practices on the use of beetle grubs as food and feed in western Kenya

**DOI:** 10.1038/s41598-023-34264-y

**Published:** 2023-05-13

**Authors:** Martin N. Wanjala, Mary Orinda, John M. Nyongesah, Chrysantus M. Tanga, Sevgan Subramanian, Menale Kassie, James P. Egonyu

**Affiliations:** 1 International Centre of Insect Physiology and Ecology (icipe), P.O. Box 30772-00100, Nairobi, Kenya; 2 Jaramogi Oginga, Odinga University of Science and Technology (JOOUST), P.O. Box 210-40601, Bondo, Kenya; 3 Present Address: Faculty of Science and Education, Busitema University, Tororo, Uganda

**Keywords:** Ecology, Zoology, Environmental social sciences

## Abstract

We examined the socio-cultural practices on the use of beetle grubs as food and feed in western Kenya by interviewing 211 randomly selected households and conducting seven focus group discussions in Bungoma, Kakamega, Busia, and Trans Nzoia counties. The grubs were used as food and feed in ~ 39% and 78% of the households, respectively. The perceived benefits of the grubs for human consumption were nutritiousness and no linkage to allergies. The grubs were perceived to enhance animal weight gain and increase poultry egg laying. They were also perceived to recycle nutrients from organic waste, and clean the environment. Toasting and roasting were the dominant methods of preparing the grubs. Lack of knowledge on the grub nutritional benefits and stigma were key deterrents to their consumption. About 66% of the respondents expressed willingness to farm the grubs if the market and rearing protocols are available. Almost 98% of the respondents lacked knowledge of the beetle biology, indicating limited capacity to conserve them. The practices on the use of beetle grubs as food and feed differed across counties and by gender, age, marital status and education level. Strategies for sustainable use of the grubs as food and feed have been proposed and new research directions highlighted.

## Introduction

The use of edible insects as part of the global food security strategy is increasingly becoming popular. About 2000 species of insects are consumed globally, ~ 500 of them in Africa, and ~ 17 in Kenya^1, 2^. Members of the order Coleoptera, commonly called beetles, account for 31% of insect species consumed worldwide^2^. These insects are mainly consumed during their larval stage, commonly called grubs. Most edible beetle larvae thrive on decomposing organic waste. Beetle grubs are rich in nutrients like protein (40.7%), fat (33.4%), energy (490.3 kcal/100 g), minerals (notably, calcium, magnesium and iron), vitamins (A, C and B 1, 2, 3 and 5) and essential amino and fatty acids^3, 4^. There is less wastage in consuming grubs than other livestock because they are consumed in their entirety once degutted, unlike 40–50% consumable portions of other livestock such as cattle, pigs and checken^3^. Insects require significantly less land, water, and feed, and release insignificant amounts of greenhouse gases compared to conventional livestock like cattle^3, 5, 6^. Adult beetles and their larvae are readily available in farmyard compost among the resource-limited rural animal keepers^7, 8^. The availability of the beetles in the community provides a wild source of start-up breeding stock for sustainable captive rearing as a source of food and feed for the future. Captive rearing as an alternative to aggressive harvesting of natural populations of the larvae could preserve them and sustain their ecological roles.

Ecologically, most beetle grubs contribute greatly to nutrient recycling through the decomposition of organic matter, parasite suppression, secondary seed dispersal and increased air permeability of the soils^8–11^. The presence of diverse gut microbes like fungi and bacteria in coprophagous beetles enhances their metabolic activity to reduce the complex components in the dung into simple organic components that are easily available for plant uptake^12^. Many adult beetles on the other hand are important crop pollinators thereby contributing immensely to increased crop productivity. For instance, scarabid beetles are important (and often obligate) pollinators of decay-scented flowers in the families Araceae and Lowiacea^9^. On the flipside, some beetles are notorious crop and animal pests, although their harvest for consumption is being advocated as a strategy for sustainably managing them^13, 14^.

According to Kusia et al.^15^, beetle grubs are the fifth most consumed insects in Kenya after termites, grasshoppers, saturniids and crickets. However, the report by these authors indicates that consumption of beetle grubs is restricted to communities in western Kenya, which may be a result of cultural differences across regions. A more in-depth investigation of sociocultural practices on the use of beetle grubs within areas where they are known to be consumed would be helpful. Whereas insects are used in many countries worldwide as food and feed, disgust towards insect consumption is still common in some communities, especially in the global west. For instance, La Barbera et al.^16^ reported that disgust toward eating insects arises because some consumers associate them with faeces, decaying matter, and other disgust-eliciting substances. Other reports attribute dislike for insect consumption to fear and discomfort, influence of western culture, limited knowledge on benefits of insect consumption, unfamiliar sensory characteristics like taste, safety concern, and their scarcity^17, 18^. Whereas various aspects of utilizing insects as food and feed have been investigated in Kenya^19^, information on socio-cultural practices by rural communities using beetle grubs as food and feed in the country is scarce.

In this study, we analysed the socio-cultural practices on the use of beetle grubs as food and feed in western Kenya, and the factors associated with these practices, with a view to exploring factors around current and potential use of grubs as a protein source.

## Results

### Socio-demographic characteristics of the respondents

The composition of respondents by gender, marital status and educational level were not significantly different across counties (Table 1). Overall, 55% of the respondents were female, while 45% were male. Most of the respondents (81.5%) were married, while the rest were either widowed or single. Most respondents had completed primary and/or secondary school levels, with (35.5%) and (32.7%), respectively. A minority (4.7%) of the respondents had attained university education. The major economic activities in the study areas were largely similar and dominated by crop farming (97.6%), poultry farming (82.9%) and cattle keeping (65.4%). Other economic activities included goat keeping, pig farming, business and formal employment, which showed significant differences across the counties. Respondents from Trans Nzoia were neither pig keepers nor formally employed and they were the least involved in goat keeping and business. Busia had the highest percentage of both goat and pig farmers. Respondents engaged in business and formal employment were commonest in Bungoma and Kakamega.

**Table 1:** Socio-demographic characteristics of respondents (%).

Characteristic	Bungoma	Kakamega	Busia	Trans Nzoia	Overall	χ^2^	*P*
Gender							
Female	45.9	54.2	67.3	54.0	55.0	5.3	0.155
Male	54.1	45.8	32.7	46.0	45.0	5.3	0.155
Marital status							
Single	11.5	6.3	3.8	6.0	7.1	2.8	0.454
Married	77.0	79.2	82.7	88.0	81.5	2.4	0.489
Widowed	11.5	14.6	13.5	6.0	11.4	2.1	0.559
Education level							
None	13.1	8.3	3.8	8.0	8.5	3.1	0.392
Primary	27.9	37.5	46.2	32.0	35.5	4.5	0.217
Secondary	29.5	31.3	42.3	28.0	32.7	3.0	0.393
Tertiary	23.0	16.7	7.7	26.0	18.5	6.8	0.079
University	6.6	6.3	0.0	6.0	4.7	3.5	0.353
Main economic activity							
Crop farming	95.1	100.0	100.0	96.0	97.6	4.7	0.194
Cattle keeping	65.6	64.6	75.0	56.0	65.4	7.0	0.321
Goat keeping	13.1	2.1	15.4	2.0	8.5	38.6	< 0.001***
Pig farming	1.6	8.3	32.7	0.0	10.4	63.6	< 0.001***
Poultry farming	86.9	81.3	78.8	84.0	82.9	4.1	0.668
Business	8.2	8.3	7.7	2.0	6.6	32.2	< 0.001***
Formal employment	4.9	4.2	1.9	0	3.3	33.6	< 0.001***

Asterisks “***” indicate significant differences at *P* < 0.001. Formal employment refers to regular earnings from an entity based on written terms of reference.

### Practices on the use of beetle grubs as food

Most respondents (96.7%) reportedly consumed at least one insect within 12 months prior to the interview, with no statistical differences across counties (Table 2). Beetle grubs (38.9%) were the second most consumed insects in the study area after termites (94.3%), with the highest beetle grub consumption rate (72.1%) recorded in Bungoma county. Of the six types of insects consumed in the area, the county of residence was only associated with consumption of beetle grubs and locust. The rate of consumption of locusts was highest in Busia. The grubs were consumed by all household members (men, women and children of either gender) alike with significant differences in percentages of each category consuming the grubs across counties.

**Table 2:** Consumption rates relative to other edible insects and category of household member, frequency of consumption, preparation methods and reasons for consuming or disliking beetle grubs (%).

Parameter	Bungoma	Busia	Kakamega	Trans Nzoia	Overall	χ^2^	*P*
Insect consumption							
At least one insect	100	96.2	95.8	94	96.7	3.4	0.338
Wild termite	93.4	98.1	93.8	92	94.3	2.0	0.575
Solitary grasshopper	1.6	5.8	0	0	1.9	9.9	0.13
Beetle grub	72.1	3.8	33.3	40	38.9	55.9	< 0.001***
Swarm grasshopper	83.3	16.7	0	0	28.6	2.9	0.286
Locust	1.6	11.5	0	0	3.3	14.9	0.002**
Crickets	1.6	3.8	0	0	1.4	3.6	0.306
Beetle grub consumption by category of household member							
Men	52.5	3.8	18.8	28	27	44.4	< 0.001***
Women	37.7	1.9	22.9	20	21.3	30.5	< 0.001***
Children ≤ 5 years	26.2	1.9	10.4	16	14.2	24.4	< 0.001***
Girls (5–18 years)	29.5	1.9	8.3	18	15.2	29.3	< 0.001***
Boys (5–18 years)	31.1	1.9	8.3	20	16.1	31.4	< 0.001***
Frequency of beetle grub consumption							
Daily during season	0	0	2.1	0	0.5	24.9	0.003**
Weekly during season	21.3	1.9	29.2	26	19.4	33.7	< 0.001***
Methods of beetle grub preparation							
Toasting	62.3	1.9	25	26	30.3	57.5	< 0.001***
Roasting	24.6	38	22.9	28	19.9	22.4	0.001**
Boiling	3.3	0.0	0.0	0.0	0.9	17.4	0.008**
Frying	3.3	0.0	2.1	0.0	1.4	16.5	0.011*
Benefits of beetle grub consumption							
Tastiness	44.3	3.8	20.8	32	26.1	40.7	< 0.001***
Nutritiousness	44.3	3.8	33.3	26	27.5	28.2	< 0.001***
Culture	6.6	0.0	0.0	2.0	2.4	19.2	0.004**
Medicinal	3.3	1.9	2.1	0.0	1.9	14.6	0.023*
Reasons for disliking beetle grubs							
Lack of knowledge	8.2	25	27.1	40	24.2	31.4	< 0.001***
Stigmatization	4.9	11.5	2.1	2.0	5.2	27.0	< 0.001***
Bad taste	3.3	1.9	4.2	0.0	2.4	23.9	0.001**
Unsafe	0.0	0.0	4.2	0.0	0.9	33.9	< 0.001***

Asterisks “*”, “**” and “***” indicate significant differences at *P* < 0.05, 0.01 and 0.001, respectively.

The grubs were consumed daily (24.9%) or weekly (33.7%) during the harvesting season which coincided with long rainy seasons. The consumption rates were however significantly different across counties. Toasting was the most common method of preparing beetle grubs (57.5%) followed by roasting (22.4%). The choice of the method of preparing beetle grubs, the benefits attributed to their consumption and reasons for disliking the grubs had significant differences across the counties.

Nutritiousness was the most popular perceived benefit of consuming the beetle grubs (27.5%) followed by tastiness (26.1%). Lack of knowledge that the grubs are edible (24.2%) and fear of stigmatization (5.2%) were the leading reasons provided as negative responses concerning consumption of grubs.

The grubs were mostly obtained from cattle dung (98.8%) with only 1.2% obtained from decomposed chicken droppings and maize stalks. Upon collection, the grubs are degutted and washed before they are prepared for consumption by boiling, toasting, roasting or frying.

### Association of age with practices on consumption of beetle grubs

With the exception of nutritiousness and culture as the reasons for consuming beetle grubs, the age of the respondent was significantly associated with all responses on reasons for consuming or not consuming beetle grubs (Table 3). Beetle grub consumption rates tended to increase with age. Approximately 2.5–3.7% of respondents aged 35 years or less consumed beetle grubs, compared to 19.8–22.2% of respondents who were 36–55 years old, and 51.9% of respondents aged 56 years and above. Among households headed by people aged 35 years and below, the grubs were only prepared by toasting. In households headed by people aged 36–45 and 46–55 years, boiling and frying were the dominant methods of preparing the grubs, respectively. Meanwhile, households headed by elderly people above 56 years mainly prepared the grubs by roasting, boiling and toasting, but not frying. Ascribing medicinal value to the grubs was only recorded among respondents younger than 25 years and those aged 56 years and above. Meanwhile, the report that the grubs are tasty increased with age from 0 to 3.6% among respondents younger than 35 years to 54.5% for those aged 56 years and above. The prominent reasons for disliking the grubs were bad taste and smell for age-group 26–35 years; safety concern and bad taste for those aged 36–45 years; and safety concern for those more than 56 years. Lack of awareness of benefits as the reason for disliking grubs was mostly reported by respondents aged 56 years and above (38%), and least by those younger than 25 years (2%). Bad taste as the reason for disliking grubs was only reported by respondents aged 26–35 years (66.7%) and 56 years and above (33.3%). The fear of stigmatization was highest among respondents aged 26–55 years (27.3%). No respondent younger than 25 years expressed a fear of stigmatization around the consumption of beetle grubs. Only the respondents in the age categories 36–45 years and above 56 years reported safety concern as the reason for disliking the grubs. The same age groups rated culture most highly (both 34.1%) as the reason for disliking beetle grubs, whereas no respondent younger than 25 years linked dislike for the grubs to culture. Half of the respondents aged 26–45 years linked non-consumption of the beetle grubs to bad smell.

**Table 3:** Socio-demographic practices and perception on beetle grub consumption (%).

Variable	Category/statistical parameter	Consumption	Preparation methods				Consumption reasons				Reasons for dislike					
			Boiling	Frying	Toasting	Roasting	Nutritiousness	Culture	Medicinal	Tasty	Unaware of benefits	Bad Taste	Fear/Stigma	Unsafe	Culture	Bad smell
Age	Under 25	3.7	0.0	0.0	4.7	0.0	3.4	0.0	50.0	0.0	2	0	0	0	0	0
	26–35	2.5	0.0	0.0	3.1	0.0	1.7	0.0	0.0	3.6	18	66.7	27.3	0	15.9	50
	36–45	22.2	50.0	33.3	25.0	21.4	24.1	50.0	0.0	20.0	28	0	27.3	50	34.1	50
	46–55	19.8	0.0	66.7	20.3	19.0	19.0	25.0	0.0	21.8	14	0	27.3	0	15.9	0
	≥ 56	51.9	50.0	0.0	46.9	59.5	51.7	25.0	50.0	54.5	38	33.3	18.2	50	34.1	0
	χ^2^	21.1	10.3	14.0	18.4	18.3	12.6	11.6	34.8	16.6	22.1	33.6	26.2	25.4	28.9	25.8
	*P*	0.010*	0.243	0.083	0.019*	0.019*	0.130	0.170	< 0.001***	0.030*	0.037*	0.001**	0.010*	0.013*	0.004**	0.011*
Gender	Male	54.1	0	66.7	56.2	54.8	43.1	25.0	50.0	41.8	64	33.3	36.4	0	68.2	50
	Female	45.9	100.0	33.3	43.8	45.2	56.9	75.0	50.0	56.9	36	66.7	63.6	100	31.8	50
	χ^2^	7.4	6.7	5.3	6.5	3.3	8.9	6.4	4.6	67.0	2.9	3.0	6.5	5.6	5.0	2.5
	*P*	0.020*	0.035*	0.072	0.039*	0.191	0.010*	0.040*	0.100	0.030*	0.409	0.387	0.091	0.134	0.172	0.469
Education	None	13.1	50.0	0.0	12.5	14.3	13.8	0.0	0.0	18.2	6	0	0	0	2.3	0
	Primary	27.9	0.0	0.0	28.1	28.6	24.1	25.0	0.0	25.5	58	33.3	63.3	50	52.3	0
	Secondary	29.5	0.0	33.3	35.9	35.7	36.2	25.0	50.0	30.9	20	0	27.3	50	27.3	50
	Tertiary	23.0	50.0	67.7	17.2	19.0	20.7	50.0	50.0	18.2	10	33.3	0	0	11.4	0
	University	6.6	0.0	0.0	6.3	2.4	5.2	0.0	0.0	7.3	6	33.3	9.1	0	6.8	50
	χ^2^	10.5	22.0	17.3	16.2	14.21	12.1	17.6	14.6	14.5	29.7	37.3	35.3	36.7	30.6	46.6
	*P*	0.030*	0.005**	0.027*	0.390	0.076	0.150	0.020*	0.680	0.070	0.003**	< 0.001***	0.001**	0.001**	0.002**	0.001**
Marital status	Married	77.0	50.0	100.0	85.9	85.7	89.7	100.0	100.0	85.5	78	100	81.8	100	84.1	100
	Single	11.5	0.0	0.0	3.1	7.1	5.2	0.0	0.00	3.6	14	0	18.2	0	9.1	0
	Widow	11.5	50.0	0.0	10.9	7.1	5.2	0.0	0.00	10.9	8	0	0	0	6.8	0
	Widower	0.0	0.0	0.0	0.0	0.0	0.0	0.0	0.00	0.0	0	0	0	0	0	0
	χ^2^	1.7	4.7	2.6	5.3	2.4	4.8	2.6	2.2	2.2	15.2	14.3	14.7	13.4	10.7	13.4
	*P*	0.640	0.580	0.860	0.510	0.880	0.570	0.860	0.890	0.900	0.870	0.112	0.099	0.144	0.296	0.144
Economic activity	Crop farming	95.1	100	100	95.3	100	96.6	100	100	98.2	98	100	90.9	100	100	100
	Cattle farming	65.6	50	100	67.2	61.9	65.5	80	75	61.8	58.8	60	45.5	50	67.4	0
	Goat farming	13.1	50	0	7.8	4.8	8.6	20	0	7.3	3.9	0	9.1	0	15.2	0
	Pig farming	1.6	0	0	1.6	2.4	3.4	0	0	3.6	11.8	100	27.3	50	15.2	0
	Poultry farming	86.9	100	100	85.9	81.0	77.6	0	75	81.8	6.3	60	63.6	0	78.3	0
	Business	8.2	0	0	4.7	7.1	5.2	25	0	1.8	7.8	40	9.1	0	13	50
	Formal employment	4.9	0	0	1.6	0	1.7	0	0	0	0	0	0	0	0	0
	χ^2^	145.8	0.2	0.2	0.2	1.8	0.3	0.4	0.2	1.1	0.8	1.2	5.7	0.9	1.3	1.2
	*P*	< 0.001***	0.898	0.918	0.921	0.413	0.856	0.835	0.918	0.568	0.667	0.55	0.059	0.352	0.532	0.559

Asterisks “*”, “**” and “***” indicate significant differences at *P* < 0.05, 0.01 and 0.001, respectively.

### Association of gender with practices on consumption of beetle grubs

The consumption of the grubs was statistically higher in households headed by male respondents than those headed by females (Table 3). A significantly higher percentage of male headed households than those headed by females prepared the grubs by toasting (56.2%); whereas boiling the grubs was only reported in female headed households. Significantly more female headed households than those headed by males considered nutritiousness (56.9%), culture (75%) and tastiness (56.9%) as the benefits for consuming the insects. The other practices on the consumption of the grubs were not statistically associated with gender.

### Association of education level with practices on consumption of beetle grubs

Consumption of beetle grubs, their preparation by boiling and frying, culture as the reason for consumption and all reasons provided by respondents for disliking the grubs were statistically associated with education level (Table 3). Consumption of the grubs increased progressively from 13.1% among the uneducated to 29.5% for those with secondary education, and then it decreased with increase in education level to only 6.6% among university graduates. Preparation of the grubs by boiling was recorded only among the uneducated (50%) and the respondents with tertiary education; whereas frying the grubs was only recorded among respondents with secondary and tertiary education levels with 33.3% and 67.7%, respectively. Culture as the reason for consuming grubs was most popular among respondents with tertiary education (50%), but attribution of culture to beetle grub consumption was non-existent among the uneducated and university graduates. Uneducated respondents were scarcely able to provide reasons for disliking the grubs. Among the educated respondents, the percentages of respondents who reported lack of awareness of benefits of consuming the grubs and culture as the reasons why they disliked the grubs decreased with the level of education from 58% and 52.3% at primary level to 6% and 6.8% among the university graduates, respectively. Bad taste as the reason for disliking the grubs was not recorded among respondents with secondary level of education; whereas respondents in the rest of the education levels reported that they disliked the grubs because of bad taste at equal percentages of 33.3%. Fear of stigma as the reason for disliking the beetle grubs was commonest among respondents with primary education (63.3%) and not recorded among those with tertiary education. Only the respondents with primary and secondary education noted safety concern as the reason for disliking the grubs, both at 50%. Meanwhile, only respondents with secondary and university education levels reported bad taste as the reason for disliking the grubs, both at 50%.

### Association of economic activity with practices on consumption of beetle grubs

Apart from consumption which differed significantly based on economic activity, the type of economic activity and marital status of the respondents had no statistical association with any practice on human consumption of the beetle grubs (Table 3).

### Use of beetle grubs as animal feed

Most of the respondents (78.2%) used beetle grubs as animal feed, with statistical differences across counties (Table 4). A majority of these were from Kakamega (89.6%) and the minority were from Trans Nzoia (66%). Beetle grubs were majorly fed to traditional poultry (75.8%), with statistical difference across counties. The use of beetle grubs as commercial poultry feed was reported the most in Kakamega (87.5%) and the least in Trans Nzoia (62%). The use of beetle grubs to feed commercial poultry and pigs differed significantly across counties with no respondent reporting their use as commercial poultry feed in Busia and as pig feed in Kakamega and Trans Nzoia. The uses of beetle grubs as pig and commercial poultry feeds were most prevalent in Busia (9.6%) and Trans Nzoia (6%), respectively.

**Table 4:** Types of animals fed on beetle grubs and their perceived benefits (%).

Variable	Bungoma	Busia	Kakamega	Trans Nzoia	Overall	χ^2^	*P*
Animals fed on beetle grubs							
As feed for any animal	73.8	84.6	89.6	66.0	78.2	12.7	0.049*
Traditional poultry feed	73.8	80.8	87.5	62.0	75.8	13.0	0.043*
Commercial poultry feed	1.6	0.0	2.1	6.0	2.4	14.2	0.028*
Pig feed	8.2	9.6	0.0	0.0	4.7	19.2	0.004**
Benefits of beetle grubs to animals							
Weight gain	19.7	38.5	47.9	36	34.6	22.3	0.001**
Increased egg production	29.5	19.2	52.1	36	33.6	24.8	< 0.001***
Reduced cost of feed	9.8	5.8	4.2	2.0	5.7	14.4	0.025*
Boosts immunity	1.6	5.8	6.3	4.0	4.3	11.7	0.068

Asterisks “*”, “**” and “***” indicate significant differences at *P* < 0.05, 0.01 and 0.001, respectively.

Apart from boosting immunity, other perceived benefits of using the beetle grubs as animal feed differed significantly across the counties. Weight gain was most and least reported as a benefit of feeding grubs to animals in Kakamega (47.9%) and Bungoma (19.7%), respectively. Feeding beetle grubs to poultry was attributed to increased egg production mostly in Kakamega (52.1%) and least in Busia (19.2%). Reduced cost of animal feed was mostly reported in Bungoma (9.8%) and least in Trans Nzoia (2%).

### Perceived important roles played by beetle grubs in the ecosystem

Overall, 57.8% of the respondents believed that beetle grubs were important in recycling nutrients as they burry themselves in the substrate, scavenging on decomposing organic matter (Table 5). This view differed significantly across counties and it was most commonly held in Bungoma (73.8%) and least commonly in in Busia (42.3%). Seventeen percent of the respondents believed that beetle grubs are important in cleaning the environment, with a significant difference in the percentages of respondents holding this view across counties. The view was most and least popular in Kakamega (27.1%) and Busia (7.7%).

**Table 5:** Roles played by beetle grub in the environment (%).

Role	Bungoma	Busia	Kakamega	Trans Nzoia	Overall	χ^2^	*P*
Recycling nutrients	73.8	42.3	50	62	57.8	19.7	0.003**
Cleaning environment	14.8	7.7	27.1	20	17.1	17.3	0.008**

Asterisks “**” indicate significant differences at *P* < 0.01.

### Knowledge of respondents on morphological differences and life cycle of beetle grubs

Forty-eight percent of the respondents knew that there were morphological differences in beetle grubs (including in adult beetles), with no significant difference across counties (Table 6). Only 2.4% could tell that beetle grubs go through egg, larvae, pupa and adult during their development, with no statistical difference across counties.

**Table 6:** Knowledge on beetle grub morphological differences and life cycle (%).

Knowledge area	Bungoma	Busia	Kakamega	Trans Nzoia	Overall	χ^2^	*P*
Different types of beetle grubs	57.4	42.3	52.1	40	48.3	4.4	0.223
Beetle grub life cycle	3.3	1.9	2.1	2.0	2.4	3.4	0.761

### Willingness to farm beetle grubs

Overall, 66.4% of the respondents expressed willingness to adopt beetle grub farming so long as there were ready market and rearing protocols. This view differed significantly across counties (χ^2^ = 14.5; *P* = 0.002), being most dominant in Bungoma (80.3%) followed by Kakamega (72.9%), Trans Nzoia (62.0%) and lastly Busia (48.1%).

### Association of socio-demographic characteristics with practices on the use of beetle grubs as feed, knowledge of their role in the environment, their biology and people’s willingness to farm the grubs

Apart from willingness to farm the beetle grubs which significantly increased with age, from 2.1% among those younger than 25 years to 35% among those aged 56 years and above, other perceptions and practices on the use of the beetle grubs as feed, knowledge of their role in the environment and their biology had no statistical association with the age of the respondents (Table 7). Similarly, marital status of the respondents only had a significant association with their willingness to farm the grubs, being highest among the married (89.3%) and lowest among the widowers (0.7%).

**Table 7:** Socio-demographic practices and perceptions on the use of beetle grubs as feed, their role in the environment, their biology and people’s willingness to farm them (%).

Variable	Variable/statistical parameter	Use as feed				Benefits as feed				Role in environment		Beetle biology		Willingness to farm
		Any animal	Traditional poultry	Commercial poultry	Pig	Weight gain	More eggs	Reduced cost	Increased immunity	Cleaning environment	Recycling nutrients	Different morphology	Lifecycle	
Age	Under 25	2.4	2.5	0	0	2.7	4.2	8.3	0	0	2.5	2.9	0	2.1
	26–35	15.2	15.6	0	10	16.4	11.3	25	0	11.1	8.2	11.8	0	10
	36–45	22.4	22.5	20	20	20.5	23.9	25	44.4	25	22.1	23.5	20	27.1
	46–55	19.4	18.8	60	20	20.5	19.7	8.3	11.1	13.9	23.8	20.6	20	25.7
	56 and above	40.6	40.6	20	50	39.7	40.8	33.3	44.4	50	43.4	41.2	60	35
	χ^2^	3.7	4.1	8.1	3.7	7.3	9.6	9.1	9.3	11.4	11.5	1.9	2.7	20.1
	*P*	0.884	0.850	0.427	0.886	0.501	0.294	0.338	0.314	0.183	0.177	0.762	0.954	0.001**
Marital status	Married	80	79.4	100	100	86.3	83.1	100	66.7	75	80.3	81.4	100	89.3
	Single	7.3	7.5	0	0	5.5	8.5	0	0	16.7	6.6	8.8	0	4.3
	Widow	12.1	12.5	0	0	6.8	7	0	33.3	8.3	12.3	8.8	0	5.7
	Widower	0.6	0.6	0	0	1.4	1.4	0	0	0	0.8	1.0	0	0.7
	χ^2^	1.8	3.8	2.5	4.4	8.3	7.3	10.2	11.0	7.1	4.2	2.7	1.4	18.5
	*P*	0.937	0.712	0.871	0.624	0.216	0.290	0.115	0.089	0.315	0.651	0.446	0.966	< 0.001***
Gender	Male	45.5	43.8	100	60	53.4	54.9	75	22.2	47.1	45.9	54.9	60	49.3
	Female	54.5	56.3	0	40	46.6	45.1	25	77.8	58.3	54.1	45.1	40	50.7
	χ^2^	2.8	0.4	6.3	1.3	3.2	4.6	4.7	3.1	0.2	0.4	7.8	1.3	3.1
	*P*	0.252	0.804	0.044*	0.534	0.204	0.099	0.098	0.207	0.909	0.837	0.005**	0.529	0.081
Education	None	10.3	10.6	0	20	6.8	9.9	8.3	11.1	11.1	8.2	9.8	0	5
	Primary	40.6	41.3	60	30	37	32.4	41.7	55.6	38.9	25.4	36.3	40	26.4
	Secondary	29.1	28.1	20	30	31.5	32.4	25	33.3	33.3	37.7	31.4	20	37.9
	Tertiary	14.5	15	0	0	17.8	16.9	16.7	0	5.6	23.8	14.7	20	25
	University	5.5	5.0	20	20	6.8	8.5	8.3	0	11.1	4.9	7.8	20	5.7
	χ^2^	21.7	21.55	26.2	29.1	23.5	27.9	19.6	20.6	12.7	19.6	6.0	5.1	29.6
	*P*	0.005**	0.006**	0.001**	< 0.001***	0.003**	0.001**	0.012*	0.008**	0.122	0.012*	0.196	0.750	< 0.001***
Economic activity	Crop farming	97.6	97.5	100	100	97.3	94.4	100	88.9	97.2	98.4	98	100	97.9
	Cattle farming	69.1	68.8	80	50	68.5	71.8	75	77.8	47.2	73.1	66.7	60	70
	Goat farming	6.1	6.3	0	0	5.5	5.6	8.3	0	0	9.8	6.9	20	9.3
	Pig farming	12.1	12.5	0	10	16.4	11.3	8.3	33.3	5.6	8.2	10.8	20	9.3
	Poultry farming	84.8	85	100	80	79.5	81.7	75	66.7	66.7	86.1	84.3	80	84.3
	Business	6.7	6.9	0	20.0	8.2	9.9	16.7	0	13.9	57.1	9.8	20	5.7
	Formal employment	3.0	3.1	0	0	2.7	4.2	0	0	5.6	2.5	4.9	20	3.6
	χ^2^	0.2	1.1	1.2	1.7	0.6	7.5	5.6	21.4	1.0	0.2	0.1	0.1	0.4
	*P*	0.997	0.29	0.544	0.437	0.737	0.023 *	0.061	< 0.001***	0.594	0.918	0.739	0.744	0.542

Asterisks “*”, “**” and “***” indicate significant differences at *P* < 0.05, 0.01 and 0.001, respectively.

Most variables were not statistically associated with gender other than the following: only the men reported feeding grubs to commercial poultry, and a significantly higher percentage of men headed households than that of those headed by women was able to recognize that there were morphological differences in the beetle grubs.

The education level of the respondents had statistical association with the use of beetle grubs as animal feed. The percentages of those who used the insects to feed any animal or traditional poultry and those who believed that this practice enhanced animal weight gain, increased poultry egg production, reduced cost of animal feed and increased animal immunity was higher among respondents with primary education than the uneducated, but subsequently decreased progressively with increase in education level. Awareness of the role of the grubs in recycling nutrients significantly increased from 8.2% among the uneducated respondents to 37.7% among those with secondary education, then decreased progressively to 4.9% among university graduates. The willingness to farm beetle grubs significantly increased from 5% among the uneducated to 37.9% among those with secondary education, then reduced progressively to 5.7% among university graduates.

Economic activities of the respondents had no significant association with the use of beetle grubs as feed, knowledge of their role in the environment, their biology and the willingness to farm the insects. The perceptions that feeding the grubs to animals increased immunity and poultry egg laying however differed statistically based on economic activity of respondents, being dominant among those involved in the main economic activities of crop farming, cattle keeping and poultry farming.

### Respondents’ opinion about health problems associated with eating beetle grub

Each respondent was asked if they had ever experienced any allergic reaction or health complication upon consuming beetle grubs, and all reported no known allergy or health complication linked to eating the grubs.

### Focus group discussion workshop

The focus group discussion participants pointed out the following points which affirm or augment responses from the household interviews:

- Eating beetle grubs is a culture of the Bukusu people in Bungoma county, and Wanga and Banyala people in Kakamega county.
- Eating beetle grubs was believed to have medicinal value and softening of the skin.
- Beetle grubs were more delicious than other insects.
- No allergy or health complication was associated with eating beetle grubs.
- The ugly appearance of beetle grubs hampered their consumption among well off people.
- Consumption of beetle grubs was associated with religion, with Islam prohibiting it, while the people in other religions considered beetle grubs to be nutritious with medicinal value.
- Beetle grubs were considered as dirty and disgusting because they breed in unhygienic substrates therefore some people feared that eating them could cause infections, leading to vomiting and stomachache.
- The beetle grubs were prepared for consumption by different methods, including boiling, sun drying, toasting and frying.
- The grubs were available in abundance in suitable substrates (mainly decomposing organic matter and cattle dung) during the long rains.

## Discussion

This study analysed socio-cultural practices on the use of beetle grubs as food and feed in four counties in western Kenya, to guide future interventions on harnessing their potential as an alternative protein source. The study households were largely homogeneous across the four study counties in terms of gender, marital status, education level and main economic activities. These findings largely corroborate data from the Kenya Population and Housing Census of 2019 about homogeneity of socio-demographic characteristics among communities in the study area^20^. The factors associated with differences in the levels of other economic activities including goat keeping, pig farming., business and formal employment across the counties remain to be elucidated.

Our data revealed that beetle grubs were the second most consumed insects in the study area after termites, with consumption levels in Bungoma county as high as 72.1%. Future studies are required to identify the different species of the beetle grubs consumed in western Kenya. The rate of consumption of the grubs in the region is ~ 13-fold higher than the previously reported national average of 3%^15^. We speculate that the high proportion of beetle grub consumers in the study area than the national consumption level could be driven by cultural differences across regions. For instances, according to data from the Kenya Population and Housing Census of 2019^20^, the Bukusu people⁠—about 1.2 million—are the dominant ethnic community in Bungoma county. The report further indicates that Kakamega county is dominated by Wanga and Banyala people. Our Focus Group discussion revealed that these ethnic groups culturally consume beetle grubs. The consumers of beetle grubs in the other neighbouring counties of Busia and Tans Nzoia could be part of these three ethnic groups who may have migrated there. More detailed studies on association of ethnicity with consumption of beetle grubs and other edible insects are warranted.

A global study involving 13 countries, where Africa was represented by South Africa, indicated that men are more willing to eat insects than women^21^. In Tanzania, eating grasshoppers is considered taboo for women and children^22^. Our data contrastingly showed that in western Kenya, the beetle grubs were consumed by all categories of household members including men, women and children of all ages. This concurs with findings from Burkina Faso, in which all species of edible insects were indifferently consumed by children, women, and men^23^. Gender-based differences in insect consumption rates therefore differ markedly across geographical divides, probably influenced by different cultural norms. The promotion of beetle grub consumption in the study area therefore has the potential to benefit all household members, irrespective of gender and age.

The beetle grubs were mainly sourced from cattle dung compost during the long rainy season. During this period, the insects were mainly consumed either daily or weekly. These findings align with the known fact that the beetle grubs specialize in breeding on cattle dung^7^. The limited availability of the grubs during dry seasons indicates a need for research on the development of protocols for rearing them artificially, including evaluating alternative substrates for their breeding and better understating of their lifecycle.

Toasting and roasting were more dominant methods of preparing the grubs for consumption than frying, with statistical differences across counties. Grub toasting and roasting were most dominant in Bungoma and Busia, respectively. These processing methods are among the most used traditional techniques of processing edible insects in Africa, to promote their safety and palatability^22–24^. The county-based differences could be attributed to differences in ethnic cultural norms, among other factors.

The respondents identified the benefits/reasons for consuming beetle grubs as nutritiousness, tastiness, culture and medicinal value, in order of dominance, with county-based statistical differences in all cases. Previous reports have also cited tastiness and nutritiousness as key enhancers of entomophagy^25, 26^. On the other hand, our data revealed that aversion to beetle grub consumption was driven by lack of knowledge of their benefits, stigma, bad taste and safety concerns, in order of dominance, with statistical differences among counties in all cases. This finding partly corroborates previous reports which enumerate fear and discomfort, disgust, influence of western culture, limited knowledge on benefits of insect consumption, unfamiliar sensory characteristics like taste, safety concern and uneven regional availability of edible insects as deterrents to entomophagy^17, 18^. Public awareness on the nutritional value of the beetle grubs and value addition to the insects through incorporating them into familiar foods like bakery products and porridge flours may enhance their acceptability among the wider population^27, 28^.

The consumption of beetle grubs was associated with age, gender, educational level and economic activity of respondents, but not their marital status. These findings partly corroborate those of Pambo et al.^29^ that age, gender and level of formal education are among the key factors influencing insect consumption. The data indicated that the younger generation was generally more averse to beetle grub consumption. Age was also associated with preparation methods used by the respondents and the reasons why they consumed or disliked the grubs. These observations could be attributed to erosion of African traditions due to exposure to westernized cultures which mostly affects the younger generation^30^. We recommend that African governments should include promoting consumption of insects such as beetle grubs in their programmes for preserving the development, documentation, preservation and dissemination of African culture among the youth, as enshrined in the African Youth Charter^31^.

The grubs were more consumed in male headed households than those headed by females, corroborating the global report that men are more willing to eat insects than women^21^. However, there is a case of beetle grubs being a staple in the diets of Aboriginal women [and children] in Australia^2^. The influence of gender on insect consumption in different cultures therefore requires more investigation.

The data showed that consumption of the grubs increased with the level of education up to secondary, but decreased with further education level to a minimum among university graduates. Knowledge about food helps people to make choices about which food to eat, considering the long-term health implications^32^. The lower the educational level of consumers, the less nutritional knowledge they have to inform their food choices^33^. The association of educational level with insect consumption can be significant or not, depending on geographical location and familiarity with entomophagy^34^. The low consumption of the grubs among respondents educated beyond secondary level could be attributed to their higher chances of obtaining formal employment which provides them with increased income to buy different types of food^35^.

The study revealed that beetle grubs were used as animal feed, mostly traditional poultry, in 78.2% of the households, with Kakamega county leading at 89.6%. These findings suggest that the community could potentially have interest and value in farming beetle grubs. The results provide an opportunity for diversification of insects promoted as ingredients in animal feed in Kenya and beyond to include beetle grubs, rather than relying on the black soldier fly^36^.

According to the respondents, the key perceived benefits of feeding beetle grubs to animals were enhanced weight gain and increased poultry egg production, with county-based statistical differences. Improvement in weight gain, egg laying and other production parameters in poultry has also been reported with several other species of insects such as meal worms, black soldier flies and cockroaches^37–39^. This is largely attributed to improvement in gut microbial communities and physical features of the animals which boost nutrient digestion and absorption^38, 40^. There are, however, concerns over microbiological safety of using live insects from compost, which need to be addressed^38, 41^. In Africa, Kenya Bureau of Standards and the Uganda National Bureau of Standards pioneered development of standards that stipulate measures for ensuring safety of insects for food and feed during production and post-harvest handling, whether farmed or semi-domesticated, including beetle grubs^42, 43^. These regulatory milestones should be emulated by other African countries and the global community at large to promote safety in the use of insects as food and feed. The farmers’ perceptions about the benefits of beetle grubs in animal productivity requires empirical validation.

About 58% of the respondents were aware that the beetle grubs play important roles in maintaining and conserving the environment through recycling nutrients in the soil and cleaning the environment through waste decomposition. This is consistent with reports that beetle grubs play several beneficial roles in the ecosystems and nutrient recycling^8, 11, 44^. However, the respondents were largely unknowledgeable about the biology of the beetles, with 97.6% unaware that the beetle lifecycle goes through egg, larva, pupa and adult stages. At adult stage, beetles commonly feed on crops, tree plants and fruits, occasionally becoming serious pests^45^. The adults seek different habitats, including a range of decomposing organic matter, to lay eggs as the food for the upcoming larvae. Community education on the beetle biology should therefore be an important component of interventions to promote their conservation and beneficial uses.

Our data show that willingness to farm the beetle grubs increased with age. As noted earlier for beetle grub consumption, this may be associated with erosion of African traditions among the younger generation due to exposure to westernized cultures^28^. The data also indicated that married respondents expressed the highest interest to farm the grubs, should there be ready market and efficient techniques for their mass rearing, compared to widowed or single respondents. This corroborates the notion that joint decision-making generally has higher positive outcomes than households that practice sole decision-making^46^.

In terms of gender, only men respondents reported that they used the grubs to feed commercial poultry. Although women are estimated to own 70% of African poultry farms^47^, this may be true for traditional poultry but not commercial poultry. Our data numerically [but not statistically] indicated that more female respondents (56.3%) fed beetle grubs to traditional poultry compared to 43.8% of the males. Several barriers such as limited access and ownership of production resources which greatly affect African women could have hampered their ability to formalise and grow their poultry businesses into commercial enterprises^48^. Our data also indicated that more men than women were able to tell morphological differences among the beetle grubs. This may be attributed to a greater educational disadvantage among women than men in Africa^49^.

The use of beetle grubs as feed and their perceived benefits to animals, their role in the environment and their biology were statistically associated with the level of education of the respondents. The perceptions increased from the uneducated to primary and/or secondary, and then dropped to a minimum with further increase in education level to the university. This could be partly attributed to the influence of education level on people’s decision making and income levels^32, 35^.

The perceptions about increased immunity and poultry egg laying when animals are fed on the beetle grubs was associated with economic activity of respondents, being dominant among those involved in the main economic activities of crop farming, cattle keeping and poultry farming. The reasons for these associations, however, need further investigations.

## Conclusions

We conclude that beetle grubs are popularly consumed in western Kenya. The grubs are also popularly used in the area as animal feed (mainly traditional poultry). The key perceived benefits of the grubs as food are nutritiousness and not being linked to any allergic reaction by consumers. Meanwhile, the use of the grubs as animal feed is perceived to be associated with enhancement of animal weight gain and increase in egg laying in poultry. In terms of benefits to the environment, the grubs are considered to help in recycling nutrients from organic waste, thereby mitigating environmental pollution. Lack of knowledge on the nutritional benefits of the grubs and stigma were key deterrents to the consumption of the grubs. Toasting and roasting were the dominant methods of preparing the grubs for consumption. Most respondents expressed willingness to farm the grubs, if the market and rearing protocols are available. Almost all the respondents lacked knowledge of grub biology, indicating their limited capacity to conserve them. The practices on the use of beetle grubs as food and feed differed across counties and by gender, age, marital status, and education level. Consumption of the grubs was most prominent in Bungoma county, and least common in Busia county. Beetle grub consumption and use as commercial poultry feed was higher in male headed households than in those headed by females. The young generation was more averse to beetle grub consumption and less willing to farm them than the older generation. The consumption of beetle grubs increased with increase in education level to secondary, then decreased with further increase in the education level to a minimum among university graduates.

### Recommendations

We recommend (i) community education and awareness creation on nutritional value, environmental benefits and biology of the beetles and their larvae to promote their conservation and acceptability; (ii) special effort to reconnect the younger generation with African traditions such as beetle grub consumption; (iii) promoting value addition on beetle grubs such as incorporating them into more appealing food products especially targeting the youth; and (iv) strengthening development and enforcement of standards on edible insects in Africa and globally to ensure their safety to consumers. Further research is required to (i) identify species of beetle grubs consumed in western Kenya and investigate their biology; (ii) empirically validate perceived benefits of beetle grubs to human and animal nutrition; and (iii) develop and disseminate protocols for captive rearing of beetle grubs.

## Methods

### Study area, household sampling and data collection

The study was conducted in Bungoma, Kakamega, Busia and Trans Nzoia counties in western Kenya during the 1st quarter of 2021. These counties were chosen because they are inhabited by communities that are known to consume beetle grubs. In addition, most homesteads in these counties practice cattle keeping, which ensures availability of farmyard manure in which the insects thrive. The enumerators were recruited from within the region to ensure common understanding of which insects are being referred to. They were pre-trained on the content of the questionnaire and ethical issues in the survey. Although the questionnaire had pre-coded potential responses and room for unpredicted responses which would be coded thereafter, the enumerators were trained to let the respondents bring up their own responses.

**Figure 1:**
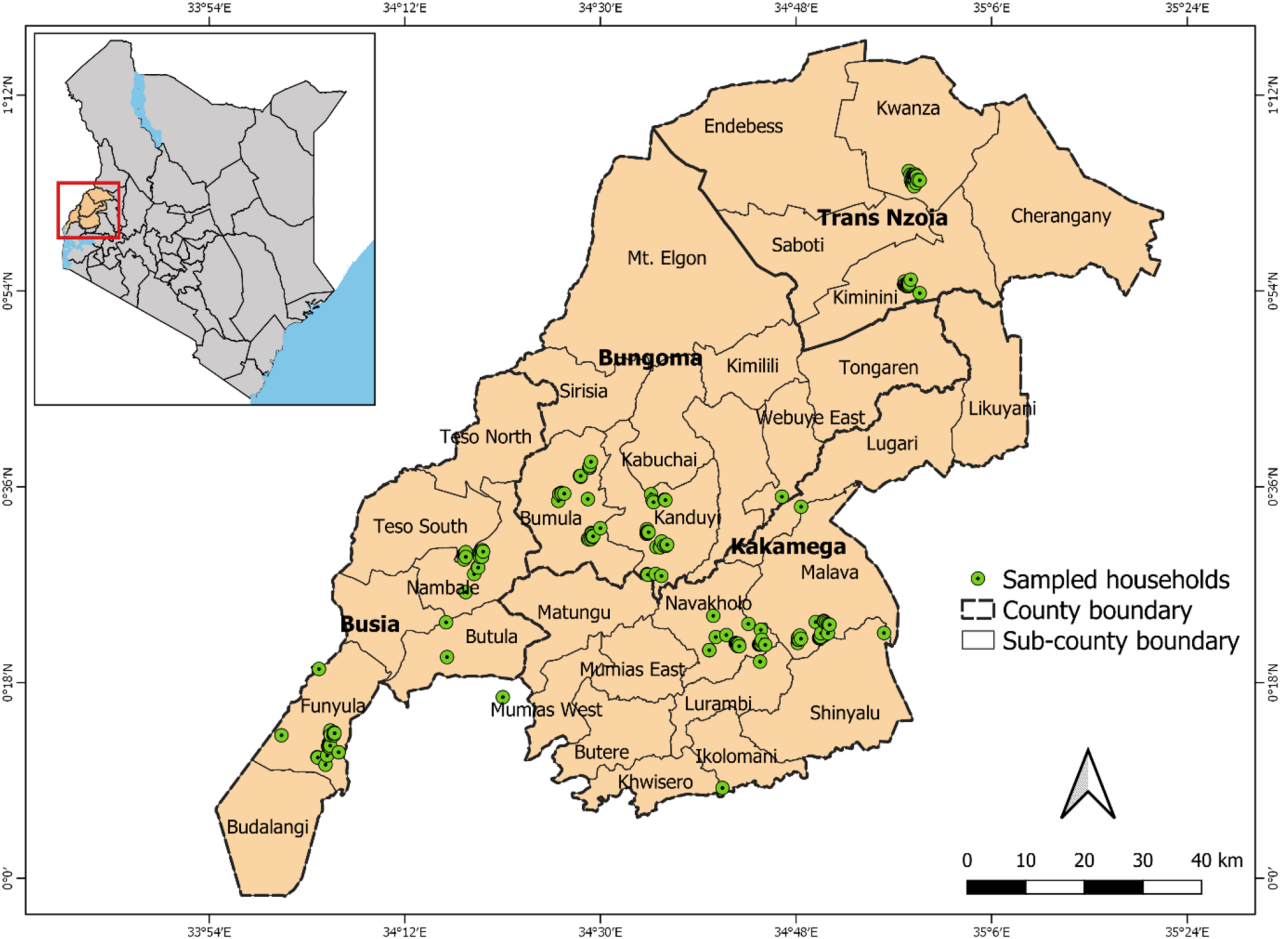
Distribution of sampled households in western Kenya. The geographical positioning system (GPS) data from the sampled households were recorded by the enumerators using GARMIN etrex 20X and used to construct a map using Arc Map QGIS 3.10.9 software (https://www.qgis.org/en/site/). Map Credit: Emily Kimathi.

A two-stage sampling procedure was used to select households from each county. The first stage involved the random selection of 5 villages within the county. In the second stage, a random sample of households within each village was selected from a village household listing developed by the first author and the survey team. Approximately 10 households were randomly selected from each village. Based on this procedure, 211 households were interviewed, distributed in the 4 counties as follows: Bungoma (*n* = 61), Busia (*n* = 52), Kakamega (*n* = 48) and Trans Nzoia (*n* = 50) (Fig. 1).

The household survey data were collected in person using a semi-structured questionnaire (Supplementary Material S1), administered by trained enumerators who spoke in both English and the local language (Swahili). The data collected included socio-demographic characteristics, knowledge, perceptions, and attitudes about beetle grubs, utilization of the insect as food or feed, perceived benefits or associated problems, and knowledge about the biology of the grubs.

The household-level data were supplemented with qualitative data from 1 to 2 focus group discussions in each county, having 10–15 participants (7 meetings with a total of 90 participants). These discussions were guided by specific questions (Supplementary Material S2). Responses from focus group discussions were not coded, but were recorded as the consensus of the participants.

### Statistical analysis

Data from household interviews were analysed using SPSS statistics software version 21 (SPSS Inc. Chicago, IL). Cross-tabulation of factors with the county was performed and Chi-square tests of independence were done at α = 0.05, to test for significant differences across counties and any relationship of responses to the demographic characteristics of the interviewees. Qualitative data from focus group discussions were not subjected to statistical analysis, but presented as consensus of the respondents.

### Ethical approval

This study was approved by the research Ethical Review Committee of Jaramogi Oginga Odinga University of Science and Technology (JOOUST), Kenya (Approval No. 7/16/1/20-30). Informed consent was obtained from all participants who were voluntarily interviewed. All methods were performed in accordance with the relevant guidelines and regulations.

## Supplementary Information

Supplementary Information 1.

Supplementary Information 2.

## Data Availability

The datasets generated and analysed during the current study are in the meantime available from the corresponding author on reasonable request.
