# Supplementary material for: Socio-cultural practices on the use of beetle grubs as food and feed in western Kenya
Source: Sci Rep. 2023 May 13;13:7805. doi: 10.1038/s41598-023-34264-y (PMC10182986; doi:10.1038/s41598-023-34264-y)
Supplement: Supplementary file 1 — Supplementary Information 1. [file 41598_2023_34264_MOESM1_ESM.doc]

**Socio-cultural practices on the use of beetle grubs as food and feed in western Kenya**

**Supplementary material S1: QUESTIONNAIRE FOR ASSESSMENT OF SOCIO-CULTURAL PRACTICES AND CHARACTERIZATION OF COMPOST GRUBS USED AS FOOD AND FEED IN WESTERN KENYA**

**Introduction**

My Name is……………. from …….. We are conducting a joint research by the International Centre of Insect Physiology and Ecology (*icipe*) and Jaramogi Oginga Odinga University of Science and Technology to assess socio-cultural practices and characterization of compost grubs or beetle larvae (hereafter referred to as compost grubs) as food and feed in western Kenya with a view to enhance their contribution to people’s nutritional status. You are among the randomly selected respondents to voluntarily participate in this research interview to get representative information for the situation in this area. This interview will take less than 30 minutes and the information collected will be kept confidentially and used anonymously for the purpose of this research only. Would you kindly let me know if you consent to participate in this interview? Yes …. No…..

PART A: SOCIO-DEMOGRAPHIC

| **Questionnaire ID (NOTE: TO BE FILLED IN ONLY BY THE ENUMERATOR) ………………….** | | | | |
| --- | --- | --- | --- | --- |
| 1.1 | Name of Enumerator |  | | |
| 1.2 | Date |  | | |
|  | **Personal** |  | | |
| 2.1 | Name of sub-location |  | | |
| 2.2 | Name of ward/constituency |  | | |
| 2.3 | Name of sub-county |  | | |
| 2.4 | Name of county |  | | |
| 2.5 | Name of household head |  | | |
| 2.5 | Name of respondent (if different from household head) |  | | |
| 2.7 | Age of household head (Years) |  | | |
| 2.8 | Marital status | Married |  | |
|  |  | Single |  | |
|  |  | Other (specify) |  | |
| 2.9 | Gender | Male = 1 | | |
|  |  | Female = 0 | | |
| 3.0 | Level of education | No education = 0 | |  |
|  |  | Primary = 1 | |  |
|  |  | Secondary = 2 | |  |
|  |  | Tertiary=4 | |  |
|  |  | University = 3 | |  |
| 3.1 | How many people have regularly lived in your/this household in the last 6 months? |  | | |
| 3.2 | ***Ask and observe*** |  | | |
| 3.3 | State and rank your main economic activities? | | | |
|  | Crop farming |  | | |
|  | Cattle keeping |  | | |
|  | Goat keeping |  | | |
|  | Pig farming |  | | |
|  | Poultry farming |  | | |
|  | Engaged in business, Specify |  | | |
|  | Formal employment |  | | |
|  | Other (Specify) |  | | |

**PART B**

1. **Compost grubs as Food**
2. Does anyone in your household consume insects? Yes …... No…..
3. If yes,

(a) List and rank all the insects that are consumed by members of your household.

| **Insect** | **Rank** |
| --- | --- |
| Compost grubs (Beetle larvae or adults) |  |
| Termites |  |
| Locusts |  |
| Crickets |  |
| Grasshoppers (Semene) |  |
| Other (Specify………………...) |  |

(b) List and rank the sources of insects consumed in your household

| **Insect** | **Rank** |
| --- | --- |
| Collected from the wild |  |
| Purchase from the community/market |  |
| Farmed insects |  |
| Other (Specify………………...) |  |

1. If farming is a source of insects consumed in your household, list and ranks all insects you farm.

| Insect | Rank |
| --- | --- |
| Compost grubs |  |
| Termites |  |
| Locusts |  |
| Crickets |  |
| Grasshoppers (*senene*) |  |
| Other (Specify………………...) |  |

1. If compost grubs are consumed in this household.
2. Give the local name(s) for compost grubs in this community……………………………
3. Tick the category of the household members who consume them?

| All=1 | Women=3 | Girls 5-18 year | Others (Specify…………..) |
| --- | --- | --- | --- |
| Men=2 | Children ≤5 years=4 | Boys 5-18 year |  |

1. If compost grubs are consumed by specific categories of members of this household, give the reasons ………………………………………………………………………………………………………………………………………………………………………………………………
2. Tick how often the members identified above consume compost grubs?

| Daily =1 | monthly =3 |
| --- | --- |
| weekly =2 | Others (Specify…………..) |

1. Give the source, frequency of buying and quantity of compost grubs?

| Source of compost grub(beetle larvae) | Frequency | Quantity (specify units e.g., Kg, number, mugs, etc and obtain relative measurements of other units in Kg) |
| --- | --- | --- |
| Cattle manure=1 |  |  |
| Compost manure=2 |  |  |
| Market=3 |  |  |
| Friend/relative=4 |  |  |
| Grass thatched straw roof=5 |  |  |

1. If from market, give frequency of purchase, quantity, and unit price.

| Frequency | Quantity (specify units e.g., Kg, number, mugs, etc and obtain relative measurements of other units in Kg) | Unit price |
| --- | --- | --- |
|  |  |  |
|  |  |  |
|  |  |  |
|  |  |  |
|  |  |  |

1. If gathered by family members, specify category and rank of the people who collect the grubs.

| Category | All | Men | Women | Children ≤5 years | Girls 5-18 year | Boys 5-18 year | Others (Specify………) |
| --- | --- | --- | --- | --- | --- | --- | --- |
| Rank |  |  |  |  |  |  |  |

1. Tick and rank the period (months) when the insects are highly available in the community

| Month | Jan | Feb | Mar | Apr | May | Jun | Jul | Aug | Sep | Oct | Nov | Dec |
| --- | --- | --- | --- | --- | --- | --- | --- | --- | --- | --- | --- | --- |
| Rank |  |  |  |  |  |  |  |  |  |  |  |  |

1. Tick and rank the procedure you use to prepare compost grubs for human consumption.

| Category | Raw =1 | Boil=2 | Fry=3 | Toast=4 | Others (Specify………) |
| --- | --- | --- | --- | --- | --- |
| Rank |  |  |  |  |  |

1. Give and rank the benefits of consuming compost grubs?

| Category | Tasty =1 | Nutritious=2 | Culture=3 | Medicinal =4 | Others (Specify……) |
| --- | --- | --- | --- | --- | --- |
| Rank |  |  |  |  |  |

1. Do you experience any allergies when these beetle larvae are consumed? Yes…. No…..
2. If yes, indicate the allergies, affected category of household members and coping methods:

| **Problem** | **Category (**All=1; Men=2; Women=3; Children ≤5=4; Girls 5-18 =5; Boys 5-18=6; Others (Specify………)) | **Method of Coping** |
| --- | --- | --- |
| Itching |  |  |
| Swellings |  |  |
| trouble breathing. |  |  |
| Abdominal pain |  |  |
| diarrhea |  |  |
| nausea or vomiting. |  |  |
| Dizziness |  |  |
| Other (Specify……………) |  |  |

j. Have you ever sold compost grubs? No=1 Yes=2

k. If yes, give frequency, quantity, and unit price of sale of compost grub?

| Quantity | Frequency (Daily, weekly, monthly, others (specify….)) | Unit price (Ksh) |
| --- | --- | --- |
| a)      less than 1kg=1 |  |  |
| b)      1kg to 5 kgs=2 |  |  |
| c)     5kgs and above |  |  |

l. If no member of your household consumes compost grub, give and rank reasons.

| Category | unaware of benefits | Bad taste | Fear stigma | Unsafe | Culture | Bad smell | Others (Specify……………) |
| --- | --- | --- | --- | --- | --- | --- | --- |
| Rank |  |  |  |  |  |  |  |

1. **Compost grub as feed**

1. Does your household use compost grub as animal feed? Yes…./No… If yes,

1. Name and rank the animals which you feed on compost grubs.

| Category | Traditional poultry | Commercial poultry | Farmed fish | Pigs | Sheep | Cattle | Goats | Others (Specify……) |
| --- | --- | --- | --- | --- | --- | --- | --- | --- |
| Rank |  |  |  |  |  |  |  |  |

(b) Tick and rank the form in which you commonly feed compost grub to the animals listed above

| Form | Rank |
| --- | --- |
| Raw =1 |  |
| Dried=2 |  |
| Grounded whole =3 |  |
| Grounded mixed with maize bran =4 |  |
| Other (specify……………………………….) |  |

**iii) Biology and ecology of compost grubs**

1. Have you noticed any differences in morphology, size, color, etc of edible compost grubs in this area? Yes…/No…

If yes, (a) describe the different types of compost grubs that are consumed in this area and give their preferred breeding substrates.

| Type description | Common breeding substrate |
| --- | --- |
|  |  |
|  |  |
|  |  |
|  |  |
|  |  |

(b) If your household members consume compost grubs, do they have any preference for the different types of the grubs? Yes……… No……….

(c) if yes to (b) describe the preferred types of compost grubs and the respective reasons

| Type description | Reason for preference |
| --- | --- |
|  |  |
|  |  |
|  |  |
|  |  |

(d)Do you know the different life stages of compost grubs? Yes…/No… If yes, name them (expected answers: egg-larva-pupa-adult; larva-larva; adult-larva) …………………………………………………………………………………………………………………………………………………………………………………………………..

(e). Have you noticed the following insects in your community (insect images which are not included in this form and were labelled A-F were shown to the respondents); where or on which plant they are, their relationship to compost grubs and their relative incidence?

| Image | Common Name in your Community | Habitat or host plant or host plant part (s) | Relationship with compost grubs (adults of compost grubs =1; common in compost with compost grub; other (specify)) | Rank |
| --- | --- | --- | --- | --- |
| A |  |  |  |  |
| B |  |  |  |  |
| C |  |  |  |  |
| D |  |  |  |  |
| E |  |  |  |  |
| F |  |  |  |  |
| Other (specify) |  |  |  |  |

(f) Rank the months based on the abundance of the insects above

| Month | Jan | Feb | Mar | Apr | May | Jun | Jul | Aug | Sep | Oct | Nov | Dec |
| --- | --- | --- | --- | --- | --- | --- | --- | --- | --- | --- | --- | --- |
| Rank |  |  |  |  |  |  |  |  |  |  |  |  |

**PART C: SOCIAL**

1. Are you a member of a farmer’s group? No=1 Yes=2
2. Are you a member of any non-agricultural groups? No=1 Yes=2
3. Are you willing to start compost grub farming as a business if market is available? No=1 Yes=2

If Yes, why?……………………………………….

1. Have you ever received any information on:
2. Compost grub production?
3. Consumption/Nutrition?
4. Market availability?
5. What is the source of information above? Tick Appropriately

| **Source** | **Score** |
| --- | --- |
| Radio=1 |  |
| Television=2 |  |
| Word of Mouth=3 |  |
| Internet=4 |  |
| Brochure=5 |  |
| Extension Officers=6 |  |
| Field days=7 |  |

1. **Please tick as appropriate, the levels at which you agree with the following statements about compost grubs as food and feed: Key: SA *=*Strongly Disagree, A*=* Agree, N*=* Neutral, D*=* Disagree SD *=*** Strongly Disagree

| **Attitude (ATT)** | **SA** | **A** | **N** | **D** | **SD** |
| --- | --- | --- | --- | --- | --- |
| Provide cheap source of protein |  |  |  |  |  |
| Rich in fats, minerals, and vitamins |  |  |  |  |  |
| Possibility of rearing guarantees availability |  |  |  |  |  |
| Rearing requires small space and is cost effective |  |  |  |  |  |
| Clean the environment |  |  |  |  |  |
| Recycle nutrients for increased crop yields |  |  |  |  |  |
| Easy to promote compost grubs as food and feed in this area |  |  |  |  |  |

| **Subjective Norms (SN)** | **SA** | **A** | **N** | **D** | **SD** |
| --- | --- | --- | --- | --- | --- |
| Compost grub is widely consumed by people in this area |  |  |  |  |  |
| Compost grubs are commonly used as animal feed in this area |  |  |  |  |  |
| There are farmer groups using compost grubs as feed in this area |  |  |  |  |  |
| Extension workers in this area encourage people to use compost grubs as food and feed |  |  |  |  |  |
| Compost grubs are widely traded in this community |  |  |  |  |  |

Please share any other information you may wish us to know about compost grubs in this area.

………………………………………………………………………………………………………………………………………………………………………………………………………………………………………………………………………………………………………………………………………………………………………………………………………………………………

Follow-up

| (We would like to have your phone number in case we have any follow-up questions. We will keep this number confidential and will only be used if we have to follow-up with something with you). | Number: _____________________________ |
| --- | --- |
| Critical observations and comments from the research assistant: | |
|
|
