# Supplementary material for: Socio-cultural practices on the use of beetle grubs as food and feed in western Kenya
Source: Sci Rep. 2023 May 13;13:7805. doi: 10.1038/s41598-023-34264-y (PMC10182986; doi:10.1038/s41598-023-34264-y)
Supplement: Supplementary file 2 — Supplementary Information 2. [file 41598_2023_34264_MOESM2_ESM.docx]

## Socio-cultural practices on the use of beetle grubs as food and feed in western Kenya

## Supplementary Material S2: Focus Group Discussion Guide

The main objective of this discussion is to **ASSESS THE SOCIO-CULTURAL PRACTICES AND CHARACTERIZATION OF COMPOST GRUBS USED AS FOOD AND FEED IN WESTERN KENYA.** This is a joint research by the International Centre of Insect Physiology and Ecology (*icipe*) and Jaramogi Oginga Odinga University of Science and Technology with a view to enhancing nutritional status of the western populace. You are among the randomly selected respondents to voluntarily participate in this research discussion to get representative information for the situation in this area. This interview will take up to 1 hour and the information collected will be kept confidentially and used anonymously for the purpose of this research only.

**Introduction:**

- Welcome of the interviewee
- Short presentation of interviewee and evaluators (name, role and title of interviewee if any)
- Short explanation of the process / evaluation
- Assurance of confidentiality (no names mentioned, coded information, summarized in the report)
- Expected time for interview (up to 1h)
- Ask for questions of the interviewee

**DISCUSSION QUESTIONS**

1. Are compost grubs utilized/consumed in this area? (Prod further form of utilization and by whom).
2. Why do you think compost grub is not popularly utilized now as compared to past years?
3. Where do you get the compost grub? (wild/compost: let them enumerate the types of compost/source of the grub/breeding substrate).
4. What are the benefits of utilizing the compost grub?
5. Any cultural connotation with utilization of the compost grub? (who is affected and reason behind it)
6. How many types of beetle/ compost grub do you know? Are they still available? (You can show them the pictures and let them identify)
7. Any noticeable/major changes then and now (seasons, availability, size, colour).
